# 3D-Printed Composites Filled with Carbon Nanotubes and Barium Titanate for Electromagnetic Applications

**DOI:** 10.3390/polym18080944

**Published:** 2026-04-12

**Authors:** Juta Varnytė, Edita Palaimienė, Jan Macutkevič, Pauline Blyweert, Aušra Selskiene, Jūras Banys, Vanessa Fierro, Alain Celzard

**Affiliations:** 1 Institute of Applied Electrodynamics and Telecommunications, Vilnius University, Sauletekio Av. 3, LT-10257 Vilnius, Lithuania; juta.varnyte@ff.stud.vu.lt (J.V.); edita.palaimiene@ff.vu.lt (E.P.); juras.banys@ff.vu.lt (J.B.); 2 IJL, CNRS, Université de Lorraine, 88000 Epinal, France; pauline.blyweert@univ-reims.fr (P.B.); vanessa.fierro@univ-lorraine.fr (V.F.); alain.celzard@univ-lorraine.fr (A.C.); 3 Center for Physical Science and Technology, Sauletekio Av. 3, LT-10257 Vilnius, Lithuania; ausra.selskiene@ftm.lt; 4 Institut Universitaire de France (IUF), 75231 Paris, France

**Keywords:** dielectric permittivity, carbon nanotubes, 3D-printed structures

## Abstract

Electromagnetic (EM) radiation emitted by various sources can cause malfunctions or damage to other electronic devices. Composite materials are widely used for EM field shielding. This work presents and analyzes the dielectric properties of 3D-printed composites containing carbon nanotubes (CNTs) and barium titanate (BaTiO_3_) over a broad frequency range. The analyzed 3D structures included a fully filled plate (PL), a basic honeycomb (BH), a honeycomb with re-entrant auxetic features (HREA), and a hierarchical honeycomb (HH). It was found that the composite material containing 1.8 wt.% CNTs and 20 wt.% BaTiO_3_ exhibits the highest absorption coefficient in the frequency range from 25 GHz to 53 GHz for all investigated 3D structures. A high concentration of BaTiO_3_ increases dielectric loss and interfacial polarization, while providing a CNT network. The synergy of these mechanisms results in the highest absorption of EM waves in the 25–53 GHz range. Moreover, all samples containing BaTiO_3_ inclusions exhibited a distinctive electrical conductivity behavior, attributed to the high complex dielectric permittivity of barium titanate, which enhances interfacial polarization. The highest conductivity and dielectric permittivity values were measured in samples containing 1.8 wt.% CNTs and 10 wt.% BaTiO_3_, while a further increase in BaTiO_3_ concentration caused a decline in dielectric performance. This effect is due to the dispersion and agglomeration of filler particles in composites with higher BaTiO_3_ concentrations.

## 1. Introduction

The rapid growth of the high-tech sector has highlighted the importance of effective electromagnetic interference (EMI) shielding to prevent it from causing malfunctions in electronic devices [1]. This is especially important for modern military and aerospace technologies, where communication security is essential. As advanced technologies become more widespread in everyday life, their negative impact on living organisms is also observed [1]. To reduce the influence of electromagnetic fields in these areas, materials capable of effectively shielding this radiation are being developed and studied [1,2,3,4,5,6,7].

Electromagnetic radiation shielding is defined as the reflection and/or absorption of a wave of a certain frequency in a material. This material is called an absorber and acts as a barrier against transmitted radiation [2]. Due to their high conductivity, metals are the most effective materials for electromagnetic shielding. However, metals are heavy, rigid, susceptible to corrosion, and difficult to process and recycle [2]. To overcome these problems, polymer and composite materials are being studied. These materials offer greater applicability due to their ease of processing, low density, and resistance to corrosion and various chemical degradations [1]. Scientific and industrial experts claim that polymer absorbers containing conductive nanoparticles give the best results [2,3]. Metals mainly shield EMI by reflection, whereas composite materials block electromagnetic radiation by absorbing it. This approach is particularly advantageous in specific applications, such as military systems and other stealth or cloaking technologies [1].

Three processes are important for shielding electromagnetic radiation: absorption, reflection, and multiple reflection. The primary mechanism of electromagnetic shielding is typically reflection at the material’s surface. This phenomenon arises from discontinuities in the electrical potential, often due to microscopic irregularities or interfacial gaps within the material [2].

The second mechanism is absorption, which is highly dependent on the material’s thickness. The shielding efficiency by absorption increases with the number of electric and magnetic dipoles that can interact with radiation in the material [4]. Materials with high dielectric permittivity can exhibit significant microwave absorption. Common examples of materials with high dielectric permittivity include barium titanate (BaTiO_3_), ferric oxide (Fe_2_O_3_), and zirconium dioxide (ZrO_2_) [5]. The absorption coefficient increases with the frequency of the EM wave, the thickness, and the conductivity of the shielding material [6].

Multiple reflections also play a significant role in electromagnetic shielding. This mechanism involves the repeated reflection of electromagnetic waves from internal surfaces, interfaces, and structural inhomogeneities within the shielding material. Materials that provide good shielding through multiple reflections usually have a large surface area or are porous. In this case, shielding occurs through repeated internal reflection of the wave within the material.

When electromagnetic radiation encounters the surface of a material, it splits into two waves: a reflected wave and a transmitted wave. This occurs because the propagation medium and the electromagnetic wave have different impedances. The amplitudes of these waves also depend on the impedances of the shielding material and the propagation medium. The wave that penetrates the material is weakened by absorption within it. The absorbed energy is dissipated as heat. When the radiation reaches the rear wall of the shielding material, part of it passes through and the rest is reflected back into the material’s volume. If the material’s thickness exceeds its absorption depth, the radiation is absorbed. Otherwise, the reflected wave undergoes multiple reflections. In this case, the shielding efficiency is greatly reduced [1].

For composite materials with hollow 3D structures, multiple reflections are particularly important, as the large surface area and voids accelerate scattering, multiple reflections, and absorption [1]. According to transmission line theory, the shielding efficiencies (*SE*) of transmittance (*T*), reflection (*R*), and absorption (*A*) can be defined as follows [1,2]:

(1) $${SE}_{T}\left(\mathrm{dB}\right)=10\;\log\frac{1}{T},$$

(2) $${SE}_{R}\left(\mathrm{dB}\right)=10\;\log\frac{1}{1-R},$$

(3) $${SE}_{A}\left(\mathrm{dB}\right)=10\;\log\frac{1-R}{T}.$$

As the efficiency level increases, the amount of energy transmitted through the material decreases. Most applications require a shielding efficiency of at least 10 to 30 dB. It has been shown that a material with a shielding efficiency of 30 dB blocks up to 99.9% of radiation. Such values are required in almost all commercial and industrial applications [7]. Other materials with SE values greater than or equal to 20 dB block about 99% of electromagnetic radiation [1].

In recent years, composite materials have been increasingly used for EM shielding. By integrating various fillers into the composite matrix, it is possible to design lightweight yet mechanically robust materials with effective electromagnetic shielding capabilities [8]. To improve shielding performance further, novel fillers such as carbon nanotubes and ferroelectric materials are being investigated, with a particular focus on their effects on the structural, electrical, and electromagnetic properties of the resulting composites [9,10,11,12]. However, achieving both reduced reflection and enhanced absorption of EM waves requires new strategies and techniques for practical implementation. Conventional manufacturing techniques for EMI shielding structures often face challenges, including high costs, limited design flexibility, and difficulties in producing intricate shapes. Over the past decade, additive manufacturing (AM) has become a widely used and rapidly expanding technology that has attracted attention for its potential as a major manufacturing technology. It can be used in various fields, such as medicine, aerospace, robotics, automotive, and construction industries [9]. 3D printing offers a promising pathway to overcome the limitations of traditional manufacturing, enabling the precise fabrication of complex parts and directly reducing waste, costs, and production time for innovative, small-scale products [13,14,15,16]. Despite this potential, the exploration of 3D printing strategies for EMI shielding materials remains relatively limited.

Traditional fully filled materials require significant resources. To address this, structured composites incorporating porous or hierarchical architectures are being developed. This approach aims to reduce material consumption and production costs while maintaining effective shielding performance without compromising mechanical robustness, stability, or thermal and chemical properties. A commonly used 3D structure is the basic honeycomb, which consists of a series of hollow cells arranged in a regular pattern and stacked in layers. This design makes them lightweight while providing strength and effective energy absorption [14]. Some studies have shown that 3D-printed composite honeycombs made from polylactic acid with graphene nanosheet-carbon nanotube fillers can achieve conductivities of up to 110.8 S/m and an EMI shielding effectiveness of 53.5 dB, surpassing the 20 dB standard for commercial shielding materials [16]. Another 3D architecture can be designed by introducing hierarchies into the basic honeycomb structure. These hierarchical structures often exhibit better overall mechanical stability and strength [13]. Two other new hybrid structures (honeycomb and re-entrant auxetic combined) were analyzed by Md. Ali et al. [14]. This group found that the newly designed hybrid structures exhibit better mechanical performance than the basic honeycomb or the re-entrant auxetic structures alone. As can be seen, various structures can replace entirely solid materials for EMI shielding applications. Accordingly, three distinct 3D architectures were chosen for this study, selected for their inherent mechanical robustness while allowing for the evaluation of their dielectric properties and EMI absorption performance. The combination of 3D-printed architectures with CNT/BaTiO_3_ hybrid composites remains relatively underexplored, particularly with respect to structure–property relationships across a wide frequency range.

## 2. Materials and Methods

Commercially available BaTiO_3_ nanoparticles (purity: 99.9%, particle size: 200 nm, tetragonal structure) and CNTs (purity: 95%, outer diameter: 10–20 nm, length: 10–30 µm), both supplied by US Research Nanomaterials (Houston, TX, USA), were used for the preparation of composites.

Hexanediol diacrylate (HDDA, SR238), pentaerythritol tetraacrylate (PETA, SR295), and acrylate oligomer (CN154 CG) were supplied by Sartomer (Arkema Group, Colombes, Île-de-France, France). Bis(2,4,6-trimethylbenzoyl)phenylphosphine oxide (BAPO) was purchased from Lambson (Arkema Group, Wetherby, West Yorkshire, England). A photocurable acrylate resin was first obtained by dissolving BAPO (0.3 wt.%) in a CN154CG/PETA/HDDA mixture (4/4/2 by weight). CNTs were then incorporated at 1.3 wt.% or 1.8 wt.% and sonicated for 20 min to ensure homogeneous dispersion within the resin. Additionally, hybrid formulations containing 1.8 wt.% CNTs combined with 10 wt.% or 20 wt.% BaTiO_3_ were prepared.

The resultant composite resins were processed using a DWS 028 J HR 3D laser stereolithography printer (405 nm laser with power 32 mW and beam diameter (resolution x/y 17 µm, layer thickness adjustable between 10 and 100 µm, DWS, Thiene, Italy) to produce four type or structures: fully filled plate (PL), basic honeycomb (BH), honeycomb with re-entrant auxetic features (HREA), and hierarchical honeycomb (HH). The structures were selected based on the design concepts reported in [13,14]. All specimens were printed with a layer thickness of 10 µm and final dimensions of 20 mm × 10 mm × 1 mm (length × width × thickness). After printing, the materials were washed in an ultrasonic bath containing isopropanol for 30 min. A UV post-treatment was performed after washing to fully complete the polymerization, as this step is essential for achieving the final properties of the printed material. The 3D structures (PL, BH, HREA, HH) were fabricated using high-resolution stereolithography (layer thickness of 10 µm, lateral resolution of 17 µm), ensuring the exact reproduction of the designed geometries. The printed samples were visually inspected and dimensionally verified to match the designed CAD models. Post-processing included isopropanol cleaning and UV curing to ensure complete polymerization.

An Elmika scalar network analyzer was used for absorption measurements. The latter were performed over a wide frequency range of 8 to 55 GHz. The waveguide was used to measure the reflection (*R*) and transmission (*T*) coefficients of the test samples. From these values, the absorption (*A*) of electromagnetic radiation could be determined using the following relationship [17]:

(4) A=1−T−R.

The microwave absorption was determined for 1 mm thick samples.

Low-frequency dielectric permittivity measurements (from 20 Hz to 1 MHz) were performed using a “Hewlett-Packard 4284A” (Hewlett, Spring, TX, USA) LCR meter. During the measurement, the sample capacitance and loss angle are recorded, from which the real and imaginary parts of the complex dielectric permittivity are then calculated. An Agilent 8714ET network analyzer (Agilent Technologies, Inc., Santa Clara, CA, USA) was used to measure a higher frequency range, from 4 MHz to 3 GHz. The network analyzer measures the signal reflection and phase. In the microwave frequency range, from 8 to 50 GHz, the reflectance and transmission of a thin dielectric rod placed inside a waveguide were studied for composite materials, while for 3D-printed structures, plate-like samples (which precisely fit the waveguide cross-section) were investigated. For these measurements, a custom-made waveguide spectrometer was used [17].

Low-temperature measurements were performed by cooling the samples at 1 K/min using liquid nitrogen. The samples were coated with silver paste to ensure contact with the electrodes.

## 3. Results and Discussion

**Figure 1:**
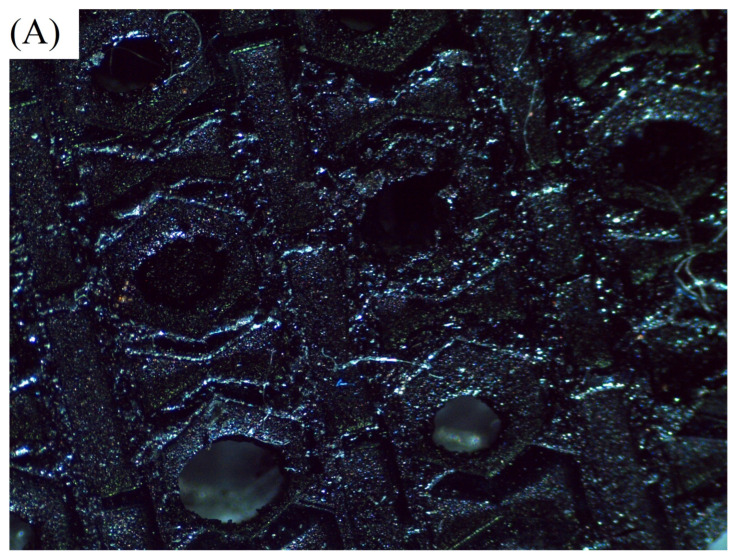

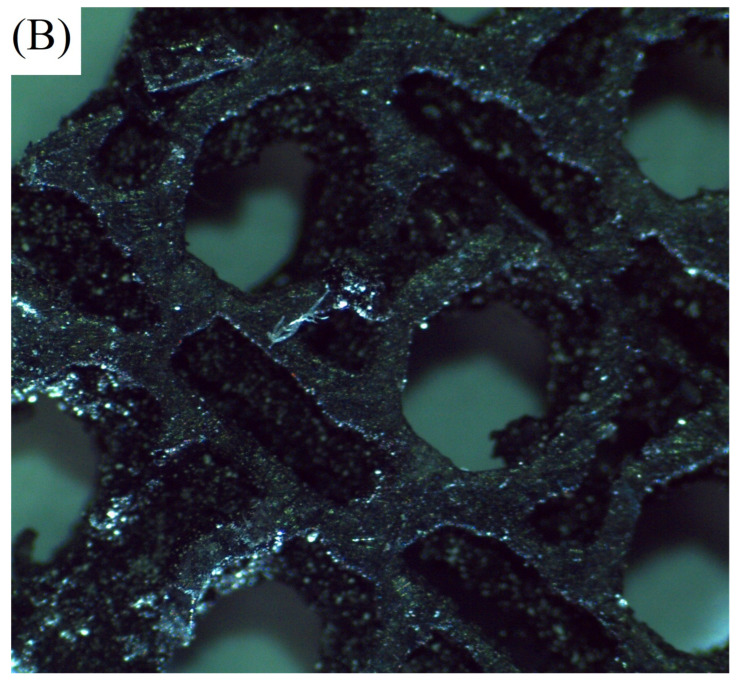
Optical photos of (**A**) 1.8% HREA structure CNTs and (**B**) 1.8% CNTs + BaTiO_3_ 20% HREA structure.

Optical photos of bulk composites and 3d-printed structures are presented in Figure 1. It can be concluded that 3d-printed structures have a good quality surface.

**Figure 2:**
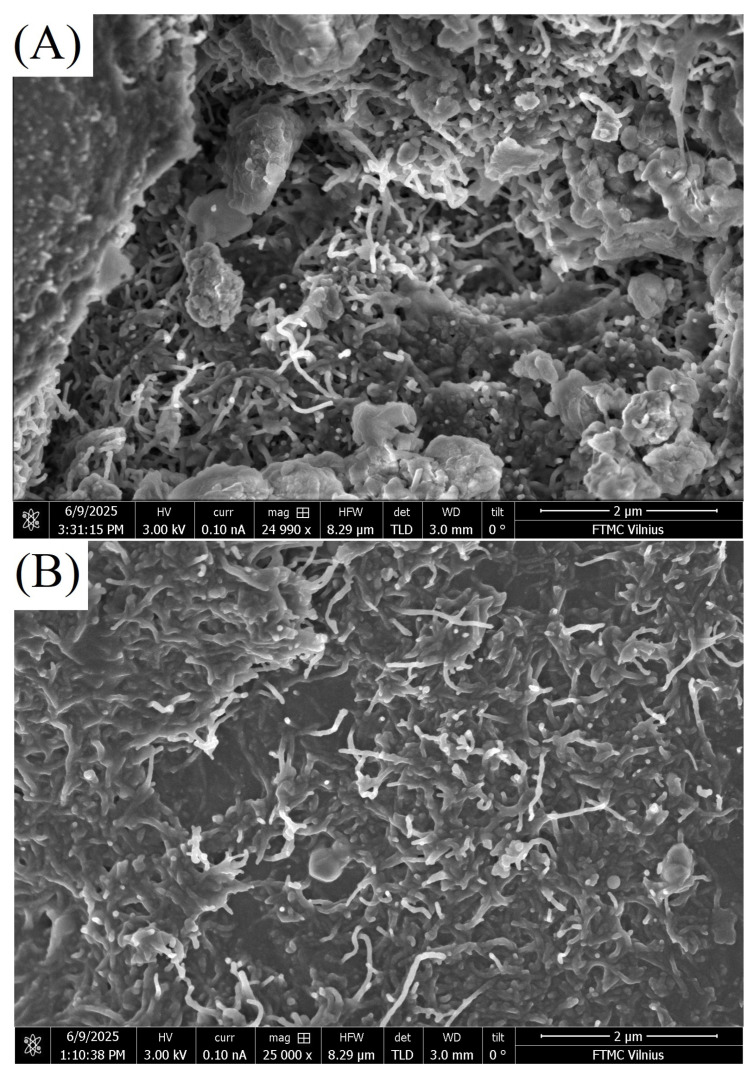

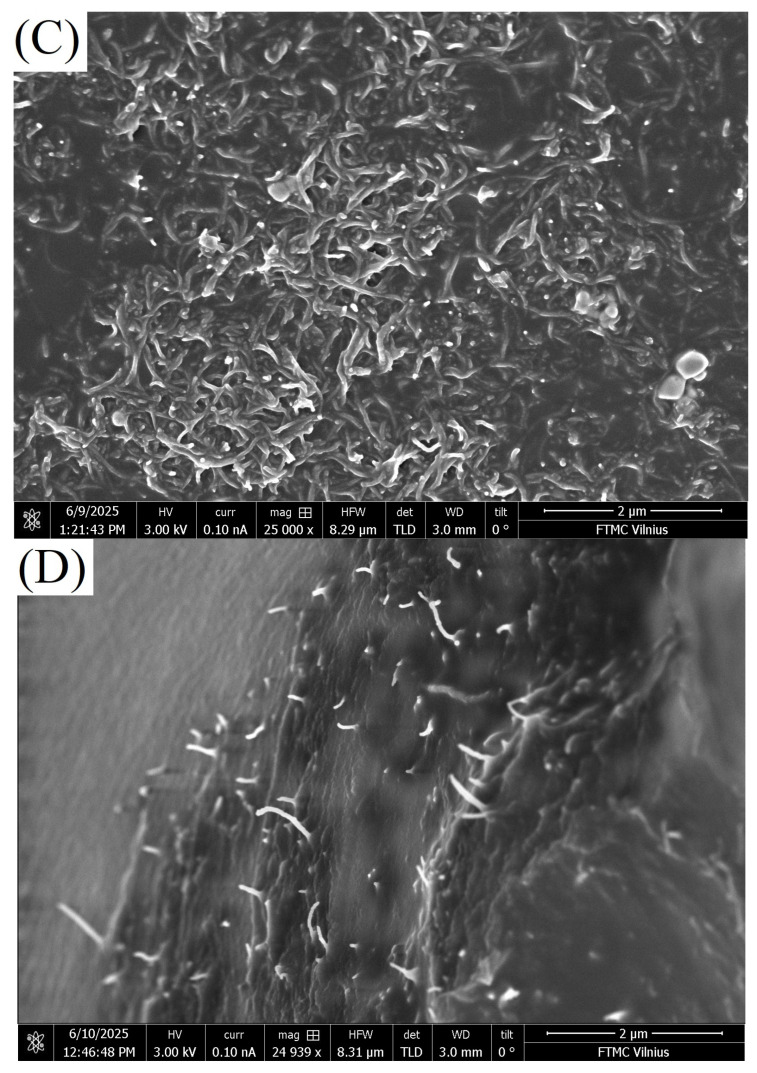
SEM images of composite samples containing (**A**) 1.8% plate CNT; (**B**) 1.8% HREA structure CNT; (**C**) 1.8% CNT + BaTiO_3_ 20%, HREA structure; (**D**) 1.8% CNT + BaTiO_3_ 20% plate illustrating their surface morphology and microstructural features.

The surface morphology of the composites was examined using scanning electron microscopy (SEM; Figure 2). A more detailed explanation has been added. The SEM analysis shows the CNTs were uniformly dispersed within the polymer matrix at both 1.3 wt.% and 1.8 wt.% concentrations, as shown by the consistent microstructure observed across all examined regions. In contrast, hybrid samples having BaTiO_3_ exhibit the formation of particle clusters, particularly at 20 wt.%, showing the onset of agglomeration. These microstructural differences are directly related to the observed changes in dielectric properties. In contrast, hybrid samples containing BaTiO_3_ exhibit the formation of particle clusters, particularly at 20 wt.%, indicating the onset of agglomeration at higher filler loadings. However, a more detailed assessment of BaTiO_3_ spatial distribution would require higher-magnification SEM and complementary EDX mapping, which were not performed in this study.

**Figure 3:**
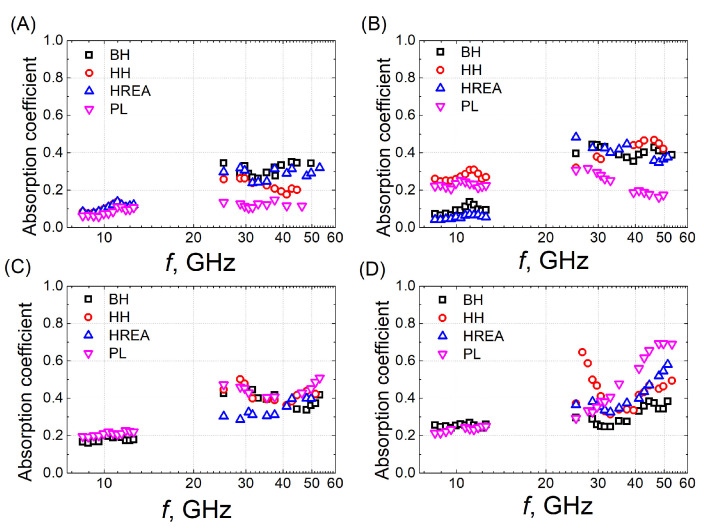
Dependence of the absorption coefficient on frequency in samples filled with (**A**) 1.3 wt.% CNTs, (**B**) 1.8 wt.% CNTs, (**C**) 1.8 wt.% CNTs + 10 wt.% BaTiO_3_, and (**D**) 1.8 wt.% CNTs + 20 wt.% BaTiO_3_.

In order to identify the composite material and 3D structure with the most effective electromagnetic radiation-shielding performance, the frequency dependence of the absorption coefficients for all prepared samples was measured (Figure 3). All measurements were conducted at room temperature. The data in Figure 3 are summarized in Table 1, which presents the maximum absorption coefficients for samples with different structures and composite compositions.

The lowest maximum absorption coefficient values (about 0.1–0.4) were measured in samples containing 1.3 wt.% CNTs for all structures. The material with a higher carbon content (1.8 wt.%) exhibits slightly improved absorption, with the maximum absorption coefficient across various 3D structures ranging from 0.3 to 0.5. Consequently, samples containing larger amounts of CNTs show enhanced absorption. This improvement is attributed to the increase in filler concentration, which facilitates the formation of additional conductive pathways, leading to denser networks and more interconnected channels. This improves the material’s electromagnetic radiation shielding properties [8].

Measurements of the absorption coefficient at low frequencies (8–13 GHz) were not performed for all samples due to limitations in fabricating specimens of the required dimensions. It was also found that at room temperature, in the frequency range from 8 GHz to 12 GHz, the absorption coefficient is low (below 0.35) and almost frequency independent. Therefore, microwave properties in the 25–53 GHz frequency range will be studied in more detail below.

The composite material containing 1.8 wt.% CNTs and 20 wt.% BaTiO_3_ exhibited the highest absorption coefficient values, which were almost independent of frequency (Figure 3C,D; Table 1). Comparison of the absorption coefficient values in Figure 3A,B indicates that the frequency response of the PL samples most closely follows that of the HREA structure. In some cases (1.3 wt.% and 1.8 wt.%), the absorption measured in the HREA structure is even higher than that of the PL samples. For these reasons, subsequent data analysis in this study focuses exclusively on HREA structure samples containing 1.8 wt.% CNTs and 20 wt.% BaTiO_3_. The HREA structure has an increased internal surface area and auxetic properties that increase multiple reflections and energy dissipation, making its properties close to those of fully filled plates.

Reducing the material density by introducing voids in the structure can decrease absorption, as electromagnetic waves are less effectively intercepted. At the same time, these voids promote multiple internal reflections within the structure. In the HREA configuration, the auxetic geometry enhances wave scattering and reduces repeated absorption. Various defects that may have been created during 3D printing can also affect the surface properties area, increasing surface polarization, scattering, and molecular polarization.

Several other factors affect the absorption of a composite material, including its composition, filler particle size, and shape of the filler particles. A higher absorption coefficient can be associated with a larger surface area and porosity of the material. In addition, the interaction between electric and magnetic dipoles in the material is important for the scattering of electromagnetic radiation [9].

**Figure 4:**
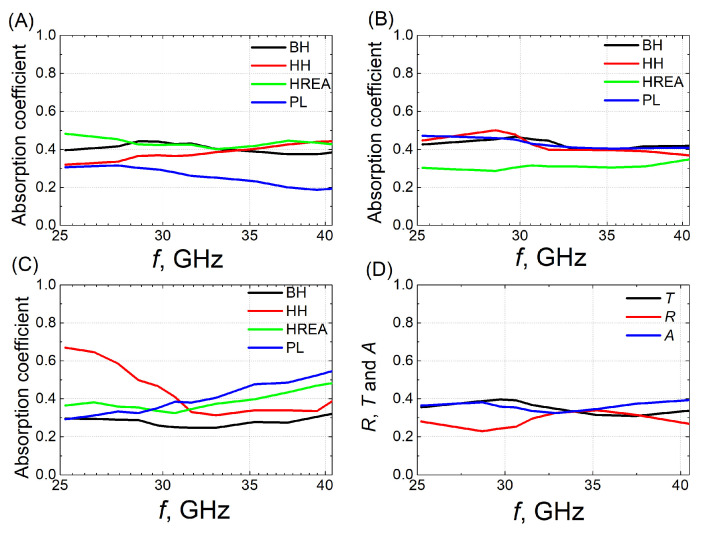
Frequency dependence of the absorption coefficient in composites with different 3D structures containing 1.8 wt.% CNTs (**A**), 1.8 wt.% CNTs + 10 wt.% BaTiO_3_ (**B**), and 1.8 wt.% CNTs + 20 wt.% BaTiO_3_ (**C**) samples. Frequency dependence of reflection, transmission, and absorption coefficients (**D**) in HREA samples with 1.8 wt.% CNTs and 20 wt.% BaTiO_3_.

In samples with 1.8 wt.% CNTs without BaTiO_3_ (Figure 4A), the highest absorption was observed in the HREA structure, reaching approximately 40 to 50%. The BH and HH structures exhibited slightly lower values, around 30–50%. The lowest absorption was measured in the fully filled plates, indicating that the structured samples provided superior absorption properties for this composition. However, when 10 wt.% BaTiO_3_ was introduced into the samples, the trend was reversed (Figure 4B). In this case, the HREA structure showed the lowest absorption, while the PL, HH, and BH structures showed higher values (~0.4). Figure 4C shows the frequency dependence of the absorption coefficient for samples with the highest concentration of CNTs (1.8 wt.%) and BaTiO_3_ (20 wt.%). The absorption coefficients measured in the different 3D structures are lower than those of the fully filled plate. In the frequency range from 30 to 40 GHz, the HREA structure exhibits higher absorption values than the HH and BH structures. A noticeable deviation is observed in the HH structure at lower frequencies: it reaches a maximum absorption of 0.7 at 26 GHz, but this peak is confined to a very narrow frequency range, after which the absorption decreases to 30–50%. Compared to samples of other compositions, the absorption coefficients of the 1.8 wt.% CNTs + 20 wt.% BaTiO_3_ composite are higher overall, which can be attributed to the high dielectric permittivity and dielectric losses of BaTiO_3_ as a ferroelectric material [10].

**Figure 5:**
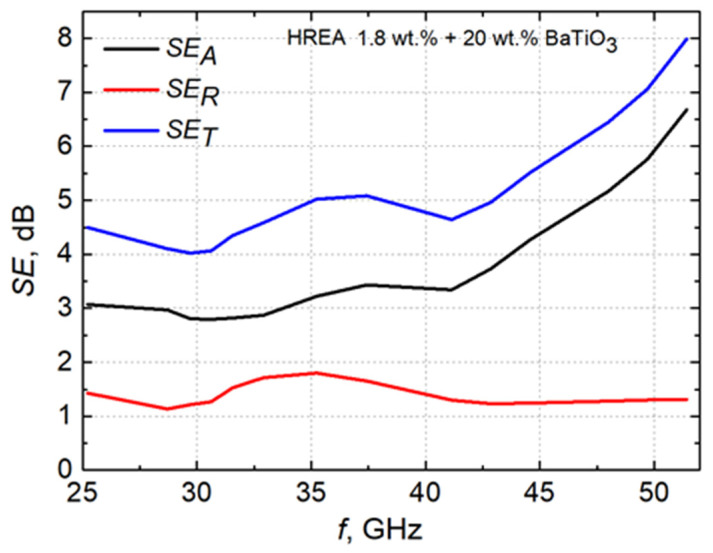
Frequency dependence of reflection, transmission, and absorption coefficients in HREA samples with 1.8 wt.% CNTs and 20 wt.% BaTiO_3_.

Figure 4D shows the dependence of reflection, transmission, and absorption coefficients on frequency for the HREA 3D structure samples with 1.8 wt.% CNTs and 20 wt.% BaTiO_3_. As the frequency increases, the transmission coefficient decreases, while the reflection coefficient remains approximately 0.3. One of the most important parameters for analyzing EM shielding properties of a material is the shielding efficiency (SE). The total efficiency (*SE*_T_) is the fraction of the incident electromagnetic wave that is intercepted. In Figure 5, we can see that this parameter in the frequency range from 25 GHz to 43 GHz is approximately 4–5 dB. At 52 GHz, the total shielding efficiency of composites with the HREA structure filled with 1.8 wt.% CNTs and 20 wt.% BaTiO_3_ exceeds 7 dB. Although the values obtained are below the level generally considered suitable for practical applications (shielding efficiency of at least 10 dB [7]), such requirements can be realistically achieved in fully filled plates, where the absence of voids in the 3D structure improves shielding performance.

Using dielectric spectroscopy, the dielectric permittivity was measured over a wide frequency range. The imaginary part *ε*″ of the dielectric permittivity data was used to calculate the electrical conductivity according to the equation:

(5) $$\sigma =2\pi \varepsilon_{0}f\varepsilon^{\prime \prime }$$

where *ω* is the angular frequency and *ε*_0_ = 8.85 × 10^−12^ F/m.

**Figure 6:**
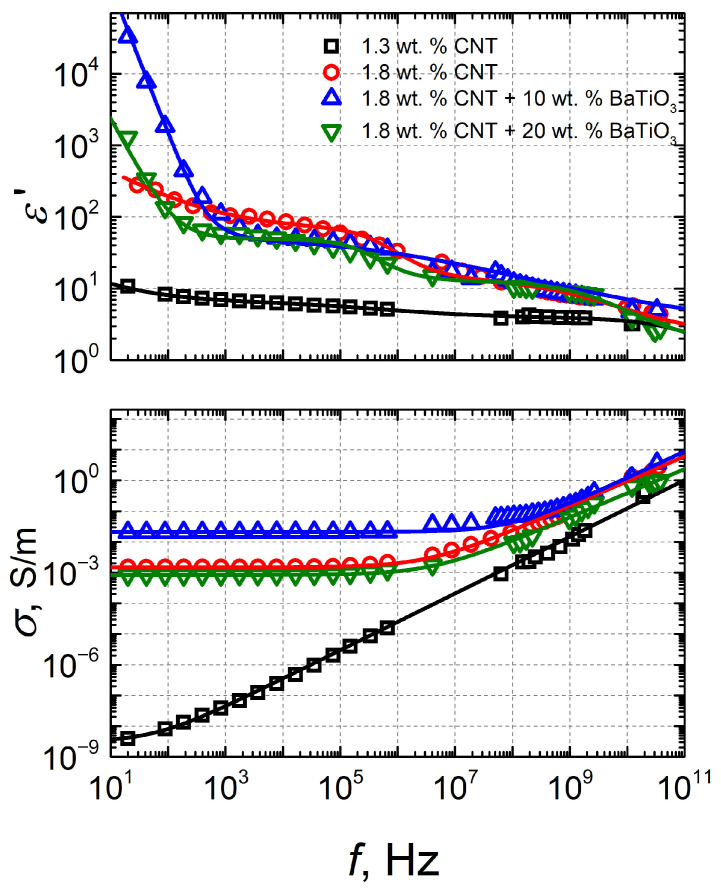
Frequency dependence of the real part of the dielectric permittivity and electrical conductivity in composites of different compositions.

Figure 6 shows the dependence of the real part *ε*′ of the dielectric permittivity and electrical conductivity *σ* on the frequency of electromagnetic radiation in composites of different compositions, at room temperature. The data are approximated by the Cole–Cole model:

(6) $$\varepsilon^{*}=\varepsilon \left(\infty \right)+\frac{\varepsilon \left(0\right)-\varepsilon \left(\infty \right)}{1+{\left(i\omega \tau \right)}^{1-\alpha }}-\frac{i\sigma }{\varepsilon_{0}\omega },$$

where *α* is the *τ* parameter describing the distribution of relaxation times (0 ≤ *α* < 1), *τ* is the relaxation time.

The obtained parameters are presented in Table 2. The Cole–Cole relaxation time almost coincides with the most probable relaxation time (Figure 6).

The Cole–Cole parameters (Table 2) reveal a clear dependence of dielectric relaxation on composite composition. For 1.3 wt.% CNTs, the relatively high *α* (0.684 ± 0.026) and *τ* on the order of 10^−9^ s indicate interfacial polarization with a moderately broad distribution of relaxation times. Increasing CNT content to 1.8 wt.% results in a further increase in *α* (0.751 ± 0.137) and a shift in *τ* to ~10^−7^ s, suggesting enhanced structural heterogeneity and slower charge relaxation due to a more developed conductive network.

The addition of 10 wt.% BaTiO_3_ maintains a high *α* (0.735 ± 0.220) and similar *τ* (~10^−7^ s), indicating that interfacial polarization remains dominant, with additional dielectric interfaces introduced by the ceramic phase. In contrast, for 20 wt.% BaTiO_3_, *α* decreases significantly (0.095 ± 0.147), pointing to a transition toward a more Debye-like (single-time) relaxation behavior, likely due to altered phase connectivity and reduced heterogeneity.

In the frequency range from 10 Hz to 40 GHz, an almost frequency-independent dielectric permittivity (*ε*′~10) was measured for samples containing 1.3 wt.% CNTs. In contrast, composites containing more CNTs (1.8 wt.%) showed a different trend: up to 1 GHz, the dielectric permittivity decreased. Both samples containing BaTiO_3_ exhibited a pronounced increase in electrical conductivity over the frequency range of 1 MHz to 100 GHz. The dielectric permittivity decreased strongly up to 1 kHz, and as the frequency increased further up to 40 GHz, the values dropped from 70 to 4 for both BaTiO_3_ concentrations. The dielectric dispersion observed at low frequencies (below 1 kHz) can be attributed to electrode polarization in conductive materials [9].

**Figure 7:**
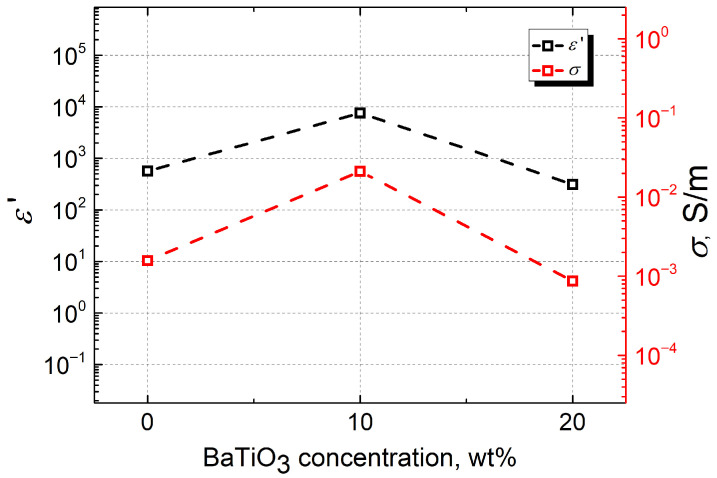
Dependence of dielectric permittivity and electrical conductivity on BaTiO_3_ concentration at 50 kHz in composites containing 1.8 wt.% CNTs.

Thus, the only materials in which no conductivity is observed are those containing 1.3 wt.% CNTs. This result can be attributed to the low filler concentration, which is insufficient to form a continuous conductive network in the composite material. Higher concentrations of conductive fillers in the composite’s polymer matrix allow for higher conductivity in the material [17]. It was found that as the CNT concentration increases, a percolation network forms, enabling efficient charge transfer. High-aspect-ratio CNTs reduce the percolation threshold and improve conductivity. The shape of the fillers is also important. Fillers with a higher aspect ratio, for example, such as tube-shaped formations, form a network for the flow of electric current more easily and efficiently than spherical particles. On the other hand, the dielectric properties of composite materials are influenced not only by the intrinsic properties and concentrations of the matrix and fillers, but also by their spatial distribution within the composite volume. Poor distribution of fillers in the polymer matrix results in low dielectric permittivity values and high dielectric losses [10]. Thus, the uniform distribution of the filler in the matrix is particularly important, since particles that agglomerate in the volume strongly affect the Maxwell–Wagner polarization. This polarization arises from charge accumulation at the interfaces between conductive CNTs and dielectric BaTiO_3_ particles. An uneven filler distribution leads to local electric-field enhancements, altering dielectric dispersion and charge-transport behavior. To facilitate data interpretation, the concentration dependence of the dielectric permittivity and electrical conductivity was plotted in Figure 7. Both parameters reach their maximum values in samples containing 10 wt.% BaTiO_3_, with the dielectric permittivity reaching 7592.35 and the electrical conductivity reaching 0.0211 S/m. The higher dielectric permittivity values in materials with a lower concentration of BaTiO_3_ can be explained by the poor dispersion and the agglomeration of fillers in materials with higher BaTiO_3_ content. It was also observed during measurements that samples with 20 wt.% BaTiO_3_ had poorer mechanical properties: they are soft and loose, and air gaps and other microcracks in the material can significantly reduce the dielectric permittivity values [10].

**Figure 8:**
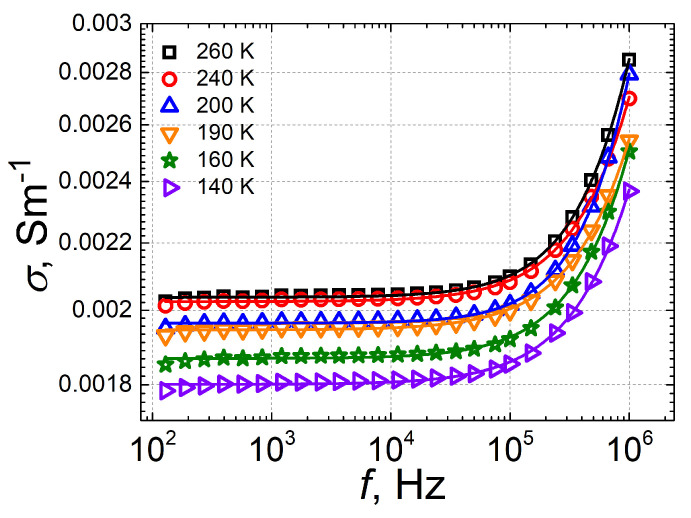
Frequency dependence of conductivity in plates containing 1.8 wt.% CNTs + 20 wt.% BaTiO_3_.

Figure 8 presents the frequency dependence of the values of electrical conductivity obtained at different temperatures for the PL sample containing 1.8 wt.% CNTs + 20 wt.% BaTiO_3_. The dispersion observed in conductivity at higher frequencies is due to the uneven hopping of charge carriers in the material, caused by the disordered distribution of impurities or defects.

The DC conductivity values were determined for each material. The resulting trends follow Arrhenius behavior, allowing the DC conductivity activation energies to be determined using Equation (7):

(7) $$\sigma_{DC}=\sigma_{0}\exp\left(-E_{A}/kT\right),$$

where *σ*_0_ is the pre-exponential constant, *E*_A_ is the activation energy, *k* is the Boltzmann constant, and *T* is the temperature [11].

**Figure 9:**
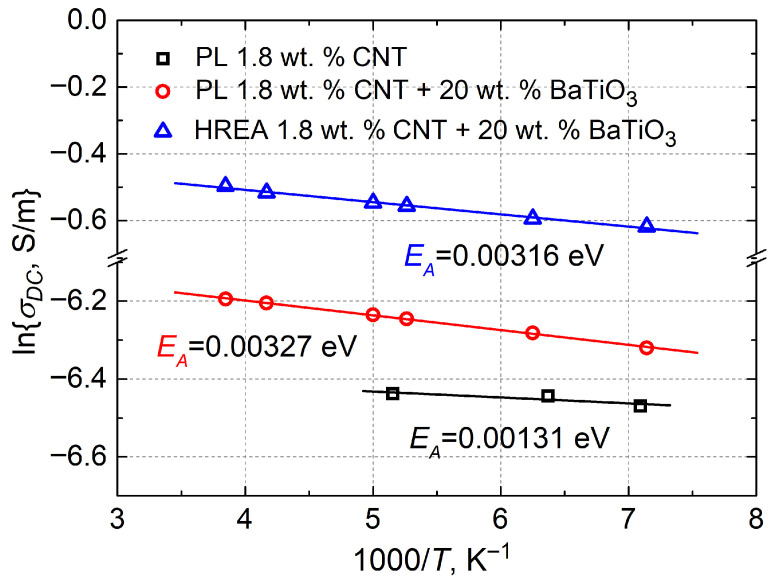
*σ*_DC_ dependence on 1000/T for plates containing 1.8 wt.% CNTs and 1.8 wt.% CNTs + 20 wt.% BaTiO_3_, and HREA structure samples containing 1.8 wt.% CNTs + 20 wt.% BaTiO_3_. The solid line is the Arrhenius fit with the conduction activation energy indicated.

The highest activation energy *E*_A_ = 0.00327 eV was calculated for a plate containing 1.8 wt.% CNTs + 20 wt.% BaTiO_3_, while a slightly lower value was obtained for the HREA structure with the same composition. The 3D structure does not affect the activation energy, as its value is determined by the material’s intrinsic properties. The activation energy values of the different structures (PL and HREA) differ only within the error limits (within 1.1 × 10^−4^ eV), which confirms the validity of the results (Figure 9).

By comparing materials with and without BaTiO_3_, it can be concluded that composites without ferroelectric components exhibit lower activation energies for DC conductivity. In samples with 1.8 wt.% CNTs + 20 wt.% BaTiO_3_, the activation energy is 2.5 times higher than in composites containing only 1.8 wt.% CNTs. Carbon nanotubes are conductive nanoparticles with a high aspect ratio. Thus, when a high dispersion of fillers in the composite matrix is achieved, a consistent network of conductive connections for charge flow is ensured. Extremely low activation energy values indicate that the material has reached the percolation threshold: the number of filler particles is sufficient for the composite to become electrically conductive [18]. This interpretation is supported by the observed flattening of *σ*_DC_ with increasing temperature, indicating the formation of an efficient charge-transport network. BaTiO_3_ particles tend to agglomerate. Thus, the addition of barium titanate fillers to such composite materials can disrupt the consistent network of conductive inclusions. BaTiO_3_ inclusions in composite materials lead to higher potential barriers, thereby increasing the activation energy.

## 4. Conclusions

This study examined the dielectric behavior and electromagnetic wave absorption of 3D-printed composites made with multi-walled carbon nanotubes (CNTs) and barium titanate (BaTiO_3_) for EMI shielding applications. The results show that the electromagnetic response strongly depends on both the composite composition and the 3D architecture. We have found that BH and HH structures reduce composite absorbance, while the HREA structure exhibits properties similar to those of the fully filled plate samples. The highest EM wave absorption coefficient in the 25 GHz to 53 GHz frequency range was achieved for composites containing 1.8 wt.% CNTs and 20 wt.% BaTiO_3_. At about 50 GHz, the total shielding efficiency of HREA structure composites with the same composition exceeds 7 dB. Although these values remain below the typical threshold required for practical EMI shielding applications (≥10 dB), and significantly below the 20–30 dB range required for most commercial and industrial applications, the present results provide important insight into the combined effects of 3D architecture and hybrid filler systems on electromagnetic performance. Moreover, all samples containing BaTiO_3_ inclusions exhibited a peculiar electrical conductivity behavior, attributed to the high complex dielectric permittivity of barium titanate, which enhances interfacial polarization. The highest conductivity and dielectric permittivity values were measured in samples containing 1.8 wt.% CNTs and 10 wt.% BaTiO_3_, while a further increase in BaTiO_3_ concentration led to a decline in dielectric performance. This effect is due to poor dispersion and agglomeration of inclusions in materials with higher barium titanate concentrations. Therefore, further optimization of the composite system is required. Possible pathways for improving shielding effectiveness include increasing CNTs content to enhance conductive network formation, optimizing BaTiO_3_ dispersion to minimize agglomeration, and designing more efficient hierarchical or multiscale 3D structures that promote absorption and multiple internal reflections. Overall, this study demonstrates that although the current SE values are not yet sufficient for most practical applications, the proposed material–structure approach provides a promising foundation for the development of lightweight, tunable EMI shielding materials.

## Figures and Tables

**Table 1:** Maximum absorption coefficient values measured in different composite structures and filler compositions.

3D Structure	Composite Composition	Maximum Absorption Coefficient	Frequency Range, GHz
PL	1.3 wt.% CNTs	0.11	34–45
	1.8 wt.% CNT	0.29	25–28
	1.8 wt.% CNTs + 10 wt.% BaTiO_3_	0.41	50–53
	1.8 wt.% CNTs + 20 wt.% BaTiO_3_	0.70	46–54
BH	1.3 wt.% CNTs	0.37	42–50
	1.8 wt.% CNTs	0.42	28–33
	1.8 wt.% CNTs + 10 wt.% BaTiO_3_	0.42	29–32
	1.8 wt.% CNTs + 20 wt.% BaTiO_3_	0.37	44–53
HH	1.3 wt.% CNTs	0.24	26–32
	1.8 wt.% CNTs	0.43	43–47
	1.8 wt.% CNTs + 10 wt.% BaTiO_3_	0.49	28–30
	1.8 wt.% CNTs + 20 wt.% BaTiO_3_	0.68	26–28
HREA	1.3 wt.% CNTs	0.30	37–53
	1.8 wt.% CNTs	0.44	25–26
	1.8 wt.% CNTs + 10 wt.% BaTiO_3_	0.40	42–50
	1.8 wt.% CNTs + 20 wt.% BaTiO_3_	0.59	50–53

**Table 2:** The best-fitted parameters to the Cole–Cole model (Equation (6)).

Samples	*α*	*τ*, *(s)*
1.3 wt.% CNTs 1.8 wt.% CNTs	0.684 ± 0.026 0.751 ± 0.137	3.837∙10^−9^ ± 1.538∙10^−9^ 3.779∙10^−7^ ± 1.35∙10^−7^
1.8 wt.% CNTs + 10% BaTiO_3_ 1.8 wt.% CNTs + 20% BaTiO_3_	0.735 ± 0.220 0.095 ± 0.147	1.00∙10^−7^ ± 1.77∙10^−7^ 6.056∙10^−7^ ± 3.54∙10^−7^

## Data Availability

The original contributions presented in the study are included in the article. Further inquiries can be directed to the corresponding author.
